# Effect of Cyclooxygenase(COX)-1 and COX-2 inhibition on furosemide-induced renal responses and isoform immunolocalization in the healthy cat kidney

**DOI:** 10.1186/s12917-015-0598-z

**Published:** 2015-12-03

**Authors:** L. Pelligand, N. Suemanotham, J. N. King, W. Seewald, H. Syme, K. Smith, P. Lees, J. Elliott

**Affiliations:** Department of Comparative Biomedical Sciences, The Royal Veterinary College, Royal College Street, London, NW1 0TU UK; Faculty of Veterinary Science, Mahidol University, Nakhon Pathom, Thailand; Novartis Animal Health Inc., Clinical Development, Basel, Switzerland; Department of Clinical Sciences and Services, The Royal Veterinary College, Hawkshead Lane, North Mymms, Hatfield, AL9 7TA Hertfordshire UK; Department of Pathology and Pathogen Biology, The Royal Veterinary College, Hawkshead Lane, North Mymms, Hatfield, AL9 7TA Hertfordshire UK

**Keywords:** Furosemide, Cyclooxygenase, COXib, NSAIDs, Robenacoxib, Ketoprofen, Feline, Renal, Macula densa, Renin-angiotensin-aldosterone system

## Abstract

**Background:**

The role of cyclooxygenase(COX)-1 and COX-2 in the saluretic and renin-angiotensin responses to loop diuretics in the cat is unknown. We propose in vivo characterisation of isoform roles in a furosemide model by administering non-steroidal anti-inflammatory drugs (NSAIDs) with differing selectivity profiles: robenacoxib (COX-2 selective) and ketoprofen (COX-1 selective).

**Results:**

In this four period crossover study, we compared the effect of four treatments: placebo, robenacoxib once or twice daily and ketoprofen once daily concomitantly with furosemide in seven healthy cats. For each period, urine and blood samples were collected at baseline and within 48 h of treatment starting. Plasma renin activity (PRA), plasma and urinary aldosterone concentrations, glomerular filtration rate (GFR) and 24 h urinary volumes, electrolytes and eicosanoids (PGE_2_, 6-keto-PGF1_α,_ TxB_2_), renal injury biomarker excretions [N-acetyl-beta-D-glucosaminidase (NAG) and Gamma-Glutamyltransferase] were measured. Urine volume (24 h) and urinary sodium, chloride and calcium excretions increased from baseline with all treatments. Plasma creatinine increased with all treatments except placebo, whereas GFR was significantly decreased from baseline only with ketoprofen. PRA increased significantly with placebo and once daily robenacoxib and the increase was significantly higher with placebo compared to ketoprofen (10.5 ± 4.4 vs 4.9 ± 5.0 ng ml^−1^ h^−1^). Urinary aldosterone excretion increased with all treatments but this increase was inhibited by 75 % with ketoprofen and 65 % with once daily robenacoxib compared to placebo. Urinary PGE_2_ excretion decreased with all treatments and excretion was significantly lower with ketoprofen compared to placebo. Urinary TxB_2_ excretion was significantly increased from baseline only with placebo. NAG increased from baseline with all treatments. Immunohistochemistry on post-mortem renal specimens, obtained from a different group of cats that died naturally of non-renal causes, suggested constitutive COX-1 and COX-2 co-localization in many renal structures including the macula densa (MD).

**Conclusions:**

These data suggest that both COX-1 and COX-2 could generate the signal from the MD to the renin secreting cells in cats exposed to furosemide. Co-localization of COX isoenzymes in MD cells supports the functional data reported here.

**Electronic supplementary material:**

The online version of this article (doi:10.1186/s12917-015-0598-z) contains supplementary material, which is available to authorized users.

## Background

The COXibs are a class of non-steroidal anti-inflammatory drugs (NSAIDs) which selectively inhibit cyclooxygenase (COX)-2, whilst sparing COX-1 activity. They were developed to provide analgesic and anti-inflammatory actions with greater safety than conventional NSAIDs, which are usually non-selective inhibitors [1]. Nevertheless, concerns over the renal safety COXibs have been raised [2]. Indeed, acute kidney injury, marked sodium retention and exacerbation of systemic hypertension have been reported with the use of COX-2 selective drugs in humans [2, 3].

COX isoforms exert key roles in the regulation of renal function through the production of protective prostaglandins (prostaglandin (PG)E_2_ and prostacyclin) [2, 4]. COX-1 is expressed in several renal cell types and is responsible for maintaining renal blood flow by attenuating vasoconstriction of the afferent arteriole (mediated by angiotensin II) through PGE_2_ or prostacyclin released locally [5]. Therefore, COX-1 inhibition by NSAIDs may precipitate ischaemic renal injury and decrease glomerular filtration rate (GFR) in hypovolaemic patients.

In several species COX-2 is constitutively expressed in a limited number of kidney cell types: the macula densa (MD) and cortical thick ascending loop and medullary interstitial cells [6, 7]. The degree of renal COX-2 expression varies with age and disease state [6, 8, 9], when renal homeostasis may become “prostaglandin dependent”. Restricted COX-2 expression suggests differing functional roles in the cortex and medulla. Indeed, prostanoids derived from medullary COX are essential in promoting natriuresis [10], whereas cortical COX regulates renin release, which maintains GFR despite fluctuations in systemic arterial blood pressure [11]. Moreover, activation of the renin angiotensin aldosterone system (RAAS) stabilises arterial blood pressure and blood volume through vasoconstriction and increases sodium and water retention.

The reliance of renin release on COX-2 was demonstrated healthy human volunteers given furosemide (high renin state model) together with celecoxib (COX-2 selective) or indomethacin (non-selective) [12]. We do not know much about effects of COX inhibition in the feline kidney or the role of each isoenzyme in mediating natriuresis and RAAS activation or on the localisation of COX expression in cats with normal kidney function. Such information has clinical safety implications, as NSAIDs are used to treat pain in cats of all ages with normal or decreased plasma volume.

The aim of the present study was to elucidate localisation of COX-1 and COX-2 and identify their roles in stimulating renin secretion, secondary to a furosemide challenge in healthy cats. Of NSAIDs licensed for cats, two have differing COX-isoenzyme selectivities: robenacoxib (1 to 2 mg kg^−1^ oral, once daily) is a selective COX-2 inhibitor [13], whereas ketoprofen (1 mg kg^−1^ oral, once daily) is a selective COX-1 inhibitor [13, 14]. The study objectives were: (1) to determine the effects of COX-2 (robenacoxib) and COX-1 (ketoprofen) inhibition on furosemide-induced changes in the healthy cats, namely (i) diuretic and saluretic responses, (ii) GFR regulation, (iii) RAAS activation and (iv) possible nephrotoxicity; (2) to identify COX-1 and COX-2 immunolocalization in kidneys from cats with normal renal function.

## Results

### In vivo furosemide study

The mean doses of robenacoxib were 1.6 mg kg^−1^ d^−1^ for once daily dosing (range = 1.38–1.74) and 3.2 mg kg^−1^ d^−1^ for dosing twice daily dosing( range = 2.76–3.36 mg kg^−1^ d^−1^).

#### Diuresis and natriuresis

**Fig. 1:**
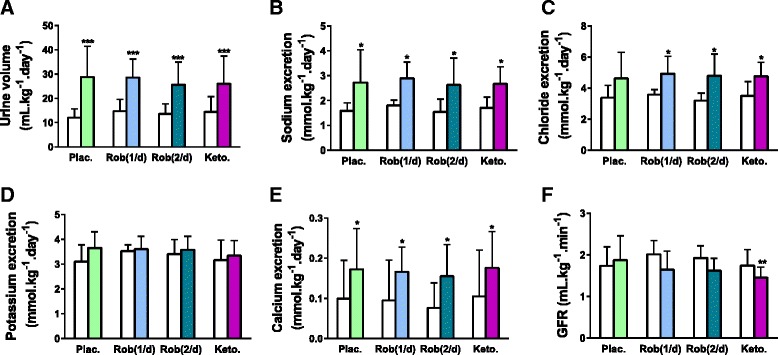
(Short title) Daily urinary volume and electrolyte excretion. (Detailed legend) Daily urinary volume (**a**), sodium (**b**), chloride (**c**), potassium (**d**), calcium (**e**) excretion and GFR determined by endogenous urinary creatinine excretion (**f**) at baseline (*white columns*) and after administration of oral furosemide (2 mg kg^−1^ twice-daily, couloured columns) with either placebo (Plac.), robenacoxib 1 to 2 mg kg^−1^ once- Rob(1/d) or twice-daily Rob(2/d), or ketoprofen once-daily (Keto, 1 mg kg^−1^). Data are presented as mean (SD) for 7 healthy cats enrolled in the crossover study. Significant change from baseline are notified as ^*^ *P* < 0.05, ^**^ *P* < 0.01 and ^***^ *P* < 0.001

Twenty-four hour-corrected urine volumes were significantly increased from baseline with all treatments, by 139, 94, 88 and 80 % with placebo, once daily robenacoxib, twice daily robenacoxib and ketoprofen, respectively (Fig. 1a). For placebo, 24 h-corrected urine output increased from 12.0 ± 3.7 to 28.8 ± 12.8 mL kg^−1^ d^−1^ (*P* < 0.001). Twenty-four hour urinary sodium excretion increased significantly in all treatment groups. For placebo, 24 h sodium excretion increased from 1.6 ± 0.3 to 2.7 ± 1.3 mmol kg^−1^ day^−1^ (*P* = 0.016, Fig. 1b). Urinary chloride excretion increased with once daily robenacoxib (*P* = 0.025), twice daily robenacoxib (*P* = 0.03) and ketoprofen (*P* = 0.03) but not with placebo (*P* = 0.07) (Fig. 1c). Urinary 24 h potassium did not significantly increase with any treatment (Fig. 1d). Urinary calcium excretion significantly increased with all treatments.

#### Haemoconcentration and GFR

**Fig. 2:**
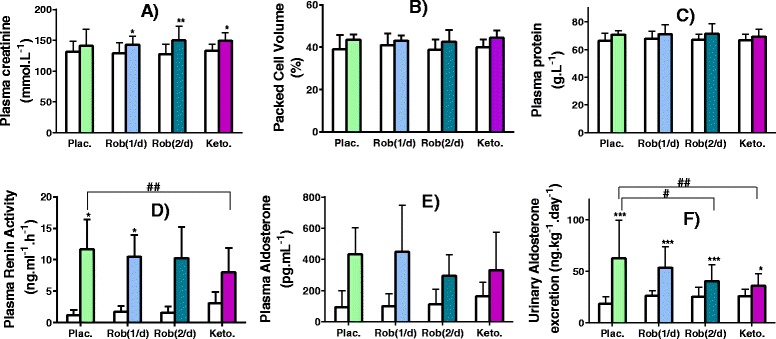
(Short title) Biochemistry and Renin Angiotensin Aldosterone system. (Detailed legend) Plasma creatinine (**a**), packed cell volume (**b**) plasma protein (**c**), plasma renin activity (**d**), plasma aldosterone (**e**) and daily urinary aldosterone excretion (**f**) at baseline (*white columns*) and after administration of oral furosemide (2 mg kg^−1^ twice-daily, *couloured columns*) with either placebo (Plac.), robenacoxib 1 to 2 mg kg^−1^ once- Rob(1/d) or twice-daily Rob(2/d), or ketoprofen once-daily (Keto, 1 mg kg^−1^). Data are presented as mean (SD) for seven healthy cats enrolled in the cross-over study. Significant changes from baseline are notified as ^*^ *P* < 0.05, ^**^ *P* < 0.01 and ^***^ *P* < 0.001. Significant differences between treatment groups are notified as ^**#**^ *P* < 0.05 and ^**##**^ *P* < 0.01

Plasma creatinine increased from baseline with once daily robenacoxib (143.1 vs 129.2 μmol L^−1^, 11 % increase, *P* = 0.029), twice daily robenacoxib (150.0 vs 127.6 μmol L^−1^, 17 % increase, *P* < 0.001) and ketoprofen (149.6 vs 133.3 μmol L^−1^, 12 % increase, *P* = 0.017) but not with placebo (Fig. 2a). For means of all treatments, there were numerically small but non-significant increases in packed cell volume (43.4 ± 0.7 % vs 39.7 ± 1.0 %, Fig. 2b) and plasma protein (69.1 ± 2.6 vs 66.7 ± 1.5 g L^−1^, Fig. 2c). A significant decrease in GFR from baseline was obtained only with ketoprofen (1.45 ± 0.25 vs 1.74 ± 0.38 mL kg^−1^ min^−1^, *P* < 0.01, Fig. 1f).

#### Effect on the renin angiotensin aldosterone system (RAAS)

Plasma renin activity (PRA) increased significantly from baseline with placebo and once daily robenacoxib but not with twice daily robenacoxib or ketoprofen (Fig. 2d). The maximal placebo-associated PRA increase was reduced by 18, 16 and 53 % with the three drug treatments, respectively. The increase in PRA from baseline was higher with placebo compared to ketoprofen (10.5 ± 4.4 vs 4.9 ± 5.0 ng ml^−1^ h^−1^, *P* < 0.01).

Plasma aldosterone concentration did not increase significantly from baseline with any treatment (Fig. 2e). However, inter-animal variability in differences between baseline and post-treatment concentrations was high. In contrast, urinary aldosterone excretion increased from baseline with all treatments (*P* < 0.001 or *P* < 0.05, Fig. 2f). The increase was greatest after placebo dosing and the magnitude of this maximal response was attenuated by 75 % by ketoprofen (*P* < 0.01) and 65 % by twice daily robenacoxib (*P* < 0.05).

#### Urinary eicosanoid excretion

**Fig. 3:**
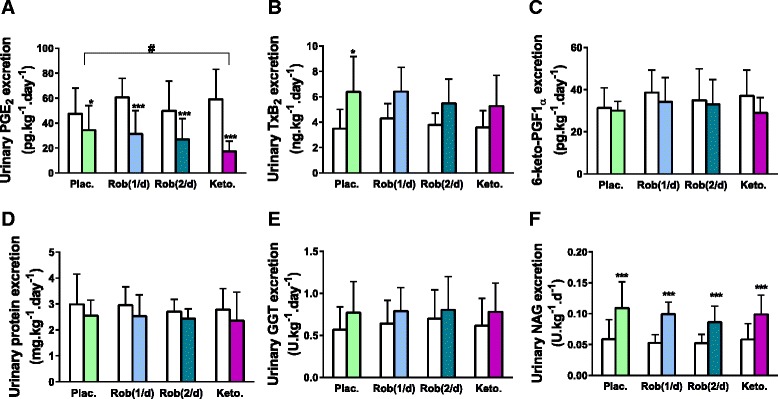
(Short title) Daily urinary prostaglandin and enzyme excretion (activity). (Detailed legend) Daily urinary prostanoids excretion [PGE_2_ (**a**), TxB_2_ (**b**) and 6-ketoPGF1_α_ (**c**)], daily proteinuria (**d**), gamma-Glutamyl transpeptidase (**e**) and N-acetyl-beta-D-glucosaminidase, NAG (**f**) urinary activities at baseline (*white columns*) and after administration of oral furosemide (2 mg kg^−1^ twice-daily, *couloured columns*) with either placebo (Plac.), robenacoxib 1 to 2 mg kg^−1^ once- Rob(1/d) or twice-daily Rob(2/d), or ketoprofen once-daily (Keto, 1 mg kg^−1^). Data are presented as mean (SD) for seven healthy cats enrolled in the crossover study. Significant change from baseline are notified as ^*^ *P* < 0.05 and ^***^ *P* < 0.001. Significant differences between treatment groups are notified as ^**#**^ *P* < 0.05

The daily urinary excretion of PGE_2_ was significantly decreased with all treatments; the relative response was greater with ketoprofen (70 ± 6.1 %) compared to placebo (27 ± 34.6 %, *P* < 0.05, Fig. 3a). PGE_2_ suppression was less and non-significant compared to placebo for robenacoxib groups (50 ± 23.0 and 47 ± 17.5 % for once and twice daily dosing, respectively). The excretion of 6-keto-PGF1α was unaffected with all treatments (Fig. 3c). Urinary TxB_2_ excretion increased significantly from baseline only with placebo (6.4 ± 2.8 vs 3.5 ± 1.5 ng kg^−1^ d^−1^, *P* < 0.021, Fig. 3b). This increase with placebo was attenuated by 27, 41 and 41 % with once daily robenacoxib, twice daily robenacoxib and ketoprofen, respectively.

#### Urinary protein and enzyme excretion

Daily urinary protein concentration and γ-glutamyl transferase activity were not increased by any treatment (Fig. 3d and e). Mean urinary N-acetyl-beta-D-glucosaminidase (NAG) activity for all treatments increased from baseline (*P* = 0.003, Fig. 3f) and there were no significant differences between treatments.

### Immunohistochemistry study

#### COX-1 expression in urinary tissues

For control tissues, strong diffuse COX-1 positive immunostaining was seen in the cytoplasm and perinuclear area in the muscle of normal feline bladders. All negative control sections had no detectable immunostaining for COX-1 and COX-2.

**Fig. 4:**
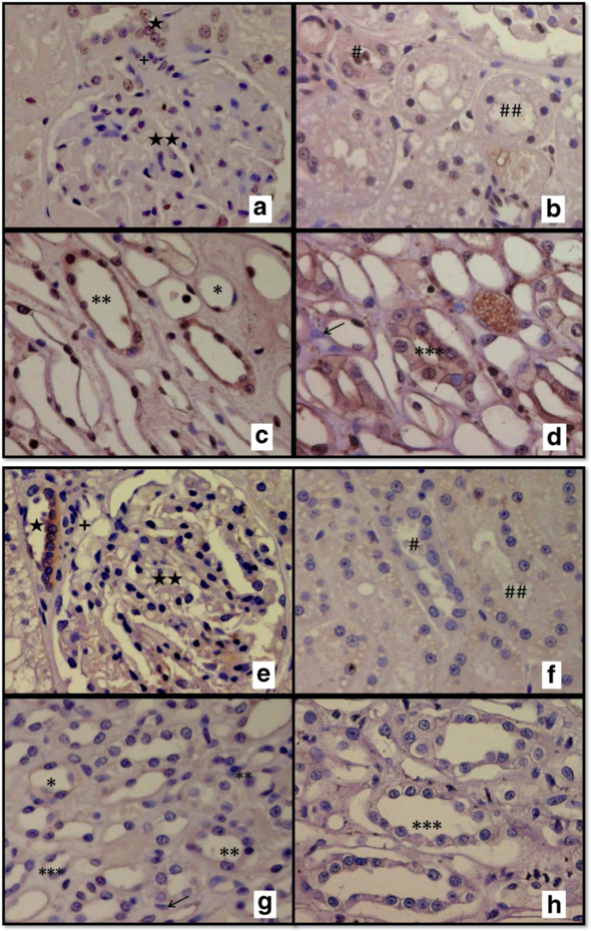
(Short title) Renal localization of COX-1 and COX-2: (Detailed legend) COX-1 (Fig. 4.a to d, 4 *top panels*) and COX-2 (Fig. 4.e to h, 4 *bottom panels*) in the normal feline kidney. Results from 21 cats (ten young and 11 old). Magnification × 400. ⋆ denotes the macula densa, ⋆⋆ denotes the glomerulus, **+** denotes the afferent arteriole, **#** denotes the distal tubule, **##** denotes the proximal tubule, * denotes the thin limb of the loop of Henle, ** denotes the thick limb of the loop of Henle, *** denotes the collecting duct and ⇽ denotes the medullary interstitial cells. **a** to **d:** The most intense expression of COX-1 was found in the thick limb of the loop of Henle (**c**) followed by the collecting duct (**d**), the distal tubule (**b**), the thin limb of the loop of Henle (**c**) and the macula densa (**a**), respectively. In the proximal tubule (**b**), the glomerulus (**a**) and the afferent arteriole (**a**), COX-1 expression was very weak to absent. **h:** The strongest COX-2 expression was found in the macula densa (**e**) followed by the thin loop of Henle (**g**), the thick limb of the loop of Henle (**g**), the proximal tubule (**f**), the medullary interstitial cells (**g**) and the collecting duct (**h**), respectively. In the distal tubule (**f**), the glomerulus (**e**) and the afferent arteriole (**e**), COX-2 was either expressed very weakly or was absent

The median [interquartile range] COX-1 expression scores of cats with normal kidneys are summarized in Table 1. The strongest immunopositive staining was observed in the thick and thin limbs of the loop of Henle and the collecting duct (Fig. 4a to d). Moderate binding of COX-1 occurred in the proximal tubule and MD. COX-1 staining was slight (weak positive in some cats and negative others) in the afferent arteriole and glomeruli in normal kidneys. COX-1 expression was undetectable in medullary interstitial cells. There was no statistically significant difference in COX-1 intensity scores between young and old cats.

**Table 1:** COX-1 and COX-2 scores in the kidney of normal young and old cats

COX-1 immunostaining	Young (*n* = 10)	Old (*n* = 11)
Thick limb	1.37 [1.01–1.66]	1.75 [1.43–1.81]
Collecting duct	1.32 [0.98–1.82]	1.41 [1.25–1.66]
Distal tubule	1.10 [0.84–1.35]	1.14 [1.00–1.43]
Thin limb	0.97 [0.58–1.20]	1.42 [0.82–1.55]
Macula densa	0.75 [0.55–0.81]	0.82 [0.80–1.13]
Proximal tubule	0.56 [0.44–0.71]	0.74 [0.36–0.91]
Glomerulus	0.48 [0.09–1.01]	0.70 [0.15–1.00]
Arteriole	0.50 [0.28–0.80]	0.60 [0.40–0.70]
Medullary interstitial cell	0.00 [0.0–0.25]	0.00 [0.00–0.00]
COX-2 immunostaining	Young (*n* = 10)	Old (*n* = 11)
Macula densa	1.29 [0.71–2.18]	1.32 [1.00–2.03]
Thin limb	1.16 [0.65–1.64]	1.10 [0.75–1.64]
Thick limb	1.10 [0.77–1.19]	0.93 [0.75–1.16]
Proximal tubule	0.73 [0.41–0.85]	1.19 [0.72–1.30]
Medullary interstitial cell	1.00 [0.00–1.00]	1.00 [0.00–1.00]
Collecting duct	0.81 [0.38–1.12]	0.90 [0.60–1.01]
Distal tubule	0.45 [0.27–1.13]	0.86 [0.42–1.09]
Glomerulus	0.75 [0.31–1.41]	0.80 [0.65–2.00]
Arteriole	0.15 [0.00–2.23]	0.10 [0.00–0.30]

For each cat, an average intensity score for each structure was calculated as described in the methods section (range 0 to 3). This single value has been used to calculate the median [interquartile range] value presented in the table for each group of cats. There was no difference in expression between young and old cats for either isoenzyme (*P* > 0.05)

#### COX-2 expression in renal tissue

Intense COX-2 positive immunostaining was seen consistently in the juxtaglomerular apparatus (JGA) of positive control sections. The regional immunohistochemical scores for COX-2 are summarised in Table 1. Positive COX-2 staining was highest in the MD, the thin and thick limb of the loop of Henle and the proximal tubule (Fig. 4e to h). COX-2 expression was usually depression was usually detectable in the glomeruli, the distal tubule and the collecting duct but only some of the arterioles. There were no statistically significant differences in COX-2 intensity scores between young and old cats.

## Discussion

This is the first study to report the acute renal effects of COX-1 and COX-2 selective NSAIDs, administered at clinically recommended doses in combination with furosemide to healthy conscious cats.

### Validation of the furosemide model

Furosemide (2 mg kg^−1^ twice daily; clinically relevant dose) produced reliable diuretic and saluretic responses and stimulated the RAAS (increased PRA and plasma aldosterone concentrations).

Non-selective and COX-2 selective NSAIDs cause sodium retention in healthy humans [10]. Moreover, non-selective NSAIDs attenuate *in vitro* the furosemide-induced reduction in loop chloride reabsorption [15]. They also compete with furosemide for the organic acid transporter in proximal tubular cells, limiting its access to NaK2Cl transporters [16]. In the present study, there was no inhibitory effect of acute NSAID administration on the diuretic and saluretic actions of furosemide; therefore electrolyte homeostasis in the cat may be regulated by mechanisms over-riding the acute COX blockade.

### Link between furosemide administration and increase in PRA

There are two possible mechanisms to explain increased renin activity in response to furosemide in the present study [17]. *First*, by blocking NaK2Cl transporter, furosemide removes the link between chloride concentration in the MD and renin secretion. Consequently, the MD signal for renin secretion persists regardless of the amount NaCl passing through the distal tubule [18], accounting for the increase in PRA. In these circumstances, basolateral cells of the MD of rabbit perfused kidneys increased COX-2 and PGE_2_ synthase expression [19, 20]. The resulting PGE_2_ release constitutes the physiological mechanism of signal transmission from MD cells to the JGA [19]. This response regulates renin release and renal vascular resistance. Using a similar experimental setting to the present study, furosemide-induced renin release was blocked by COX-2 inhibitors in humans [12]. *Second*, the later occurring action of furosemide (due to the induced sodium and water depletion) is to reduce stimulation of the afferent arteriolar stretch receptors and carotid / aortic sinus baroreceptors, thus stimulating renin release [12, 17].

### Effects of NSAIDS on RAAS

Ketoprofen significantly attenuated the furosemide-induced ten-fold increase in PRA in in this study, whereas the effect of robenacoxib was equivocal and probably dose dependent. An unavoidable limitation of the study is that ketoprofen (at the clinical dose rate and route selected) inhibits not only COX-1 for 24 h, but also COX-2 for 10 to 11 h after oral administration (see integration of pharmacokinetic and pharmacodynamic data in Additional file 1: S2). Hence, attenuation of the PRA response may be due to inhibition of COX-1 or requires inhibition of both isoforms. Inhibition of COX-2 alone was insufficient to suppress this response. To elucidate the relative importance of COX-1 and COX-2 in mediating the PRA response to furosemide administration requires the use of a highly selective COX-1 inhibitor; no such compound is currently available.

Aldosterone release from the zona glomerulosa of the adrenal cortex is controlled by angiotensin II in circumstances of renal hypo-perfusion and increase in plasma potassium. Plasma potassium concentration did not change significantly in this study (results not shown), although the fractional excretion of potassium increased with all treatments, commensurate with the decrease in GFR. Plasma aldosterone concentration fluctuates over the course of the day, as aldosterone release can be affected by several factors, such as adrenocorticotropic hormone (ACTH), body position, hyponatremia and atrial natriuretic peptide [21]. In the present study, the minor stress associated with restraint and blood sampling could have exerted an acute effect on ACTH and plasma aldosterone concentrations. The high inter-animal variability could account for the lack of significance of the numerical increase in plasma aldosterone after any of the treatment. Aldosterone excretion in urine collected over 24 h is, therefore, likely to provide a better indication of time-averaged aldosterone release than spot plasma aldosterone concentration measurement. Urinary aldosterone response was suppressed by both ketoprofen and twice daily robenacoxib and to a lesser extent by once daily robenacoxib. This supports a role for COX-2 but suggests that COX-1 is also involved in aldosterone secretion, as ketoprofen inhibited urinary aldosterone more than either dose of robenacoxib. Furthermore, the urinary aldosterone data are consistent with the PRA data, supporting the view that inhibition of both COX-1 and COX-2 is required to block the effect of furosemide in activating RAAS.

### Immunochemistry evidence for COX-1 and 2 in furosemide-induced PRA response

Evidence for COX-2, and potentially also for COX-1, involvement in signalling from the MD cells to the renin secreting cells of the JGA, was obtained by immunohistochemistry, which demonstrated high level expression of COX-2 and weak expression of COX-1 within the MD of both young and older adult cats. In other species, the use of immunohistochemistry to localise COX-isoforms has demonstrated species and age-related differences in expression. Only COX-2 has been localised to the MD in most species, including dogs, rabbits and rats [7, 22, 23], but with both isoforms identified in mice [24]. Distribution of the isoenzymes in the other compartments of the feline kidney is similar to that seen in rodents, rabbits and dogs [7, 22, 23, 25]. In humans, COX-2 expression is reported in the MD in the foetus [26] and in subjects over 50 years of age [9], hence the inclusion of two age groups of cats in the present study. Kidney tissue was obtained from cats undergoing post-mortem examination for reasons other than CKD. These cats cannot, therefore, be described as healthy, and other features of their disease process might have altered renal tissue COX-enzyme expression. Nevertheless, the data indicate that COX-2 is the principal isoform present in cells of the feline MD, whilst the finding of weak expression of COX-1 at this location suggests that interaction between the two isoforms may generate the signal to the JGA cells from the MD to secrete renin in some cats. This interpretation of the immunohistochemistry data is strengthened by the results of the functional studies which suggest involvement of both COX isoenzymes in signalling from MD to JGA to stimulate renin secretion.

### Vascular effects of furosemide and NSAIDs

In the placebo group, the significant increase in plasma creatinine and slight but non-significant increases in packed cell volume and total protein suggest contraction of the volume of the central compartment by furosemide [27]. This may have led to reduced tonic stimulation of either the stretch receptors of the afferent arteriole or the cardiac or arterial baroreceptors, leading to release of renin, angiotensin II and aldosterone. However, systolic arterial blood pressure was not measured and therefore a baroreceptor mechanism cannot be confirmed. Altered stretch of afferent arteriole wall activates RAAS and the release of angiotensin II and aldosterone, leading to increased sodium reabsorption. Offset of the vasoconstrictive action of angiotensin II on the afferent arteriole and preservation of GFR is dependent on the local release of prostaglandins [4, 28]. GFR was significantly decreased with ketoprofen and approached significance for robenacoxib (both doses); it is therefore possible that the COX-1 dependent RAAS response to volume depletion was blunted with ketoprofen, accounting for the lesser effect of robenacoxib on furosemide-induced PRA increase.

### Effects of furosemide and NSAIDs on urinary eicosanoids

Urinary eicosanoid excretion derives from local renal production. The effect of furosemide on urinary PGE_2_ excretion varies with species. It was unchanged in humans [12] but increased in dogs [29]. Surprisingly, in the present study furosemide reduced PGE_2_ excretion, probably through inhibition of PGE_2_ production. Against this background, it was not possible to relate PGE_2_ inhibition by NSAIDs to their action on furosemide-induced renin secretion. However, ketoprofen further reduced urinary PGE_2_ excretion, possibly indicating that renal COX-1 is the major source of urinary PGE_2_ in the cat. Attenuation of the furosemide increased urinary TxB_2_ excretion by ketoprofen and twice daily robenacoxib, compared to placebo, indicates that furosemide-induced renal TxB_2_ synthesis is probably the consequence of a COX-2 mediated compensatory vasoconstrictive mechanism.

### Effects of furosemide and NSAIDs on urinary protein excretion

In dogs, increase in NAG index is associated with renal proximal tubular necrosis [30]. Furosemide significantly increased urinary NAG activity in all groups in the present study and the increases were not exacerbated by concomitant NSAID treatment. For all cats, NAG returned to baseline levels during the intervals between periods, indicating either a transient nephrotoxic effect of furosemide or, more likely, an increased lysosomal turnover in response to the increase in tubular fluid flow [31, 32].

## Methods

### In vivo study design

Seven neutered experimental cats (four males, three females, 2 years old), weighing 3.7 ± 0.34 kg, were enrolled in a four period crossover study. They were group housed except during sampling periods when they were individually housed in cages equipped with a timed urine collection system previously validated in cats [33]. Each cat was fed 25 g of a low sodium dry food twice daily (RF23, Royal Canin, Aimargues, France). Drinking water was available *ad libitum*. The study complied with the EU Directive 2010/63/EU (Project License Number 70/6132) and the protocol was approved by the Royal Veterinary College Ethics and Welfare Committee. All cats were declared healthy at the end of the study and subsequently re-homed.

**Fig. 5:**
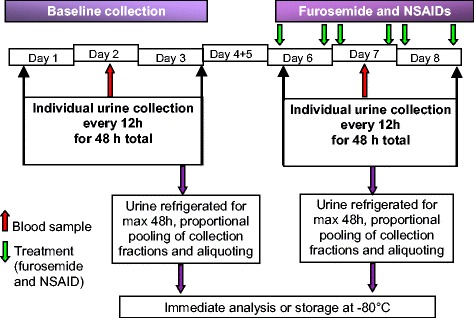
(Short title) Sampling schedule (one period of the study), including baseline and the treatment timescale. (Detailed legend) A previously validated collection system was used for urine collection on Day 1 to Day 3 and Day 6 to Day 8 [33]. The system recorded accurately the time urination occurred. Oral furosemide (2 mg kg^−1^) was administered twice-daily from the morning of day 6 to the morning of day 8. The treatment consisted of administering furosemide with one of four oral NSAID/placebo dosing regimens (placebo, 1 to 2 mg kg^−1^ robenacoxib once-daily, 1 to 2 mg kg^−1^ robenacoxib twice-daily and ketoprofen 1 mg kg^−1^ once-daily) from the morning of day 6 to the morning of day 8

At the beginning of each period, a pooled baseline urine sample was collected over 48 h during days 1, 2 and 3 (Fig. 5). A baseline blood sample (cephalic or jugular venepuncture) was collected in the middle of the urinary collection interval (between 10.30 and 11.45 am on day 2). The treatments comprised administration of furosemide 2 mg/kg (Furosemide, Milpledge, Retfort, UK) orally twice daily (08.00 am, 07.00 pm) from the morning of day 6 to the morning of day 8, concomitantly with one of four NSAID/placebo dosing regimens (Fig. 5): (1) robenacoxib (Onsior, 6 mg flavoured tablets, Novartis Animal Health, Camberley, UK,1 to 2 mg kg^−1^) once daily; (2) robenacoxib (1 to 2 mg kg^−1^) twice daily; (3) ketoprofen once daily (Ketofen, 5 mg tablets, Merial, Harlow, UK, 1 mg kg^−1^); and (4) placebo once daily (active ingredient excluded). Robenacoxib tablets composition was 6.25 % (6 mg) active ingredient, 30.0 % microcryst. cellulose Avicel PH 101, 3.0 % polyvinylpyrrolidone Kollidon K30, 43.75 % yeast, 3.0 % Crospovidone Kollidon CL, 12.0 % yeast, 0.5 % vanilla, 1.0 % colloidal silicon dioxide (Aerosil 200), 0.5 % magnesium stearate, makes a total weight of 96.0 mg per tablet.

Furosemide, NSAIDs or placebo tablets were inserted in a small cube of moist cat food to facilitate oral administration. The rest of meal was withheld until at least 90 min after drug administration. Treatment order was randomised, following a Latin square design.

Further urine collection commenced after the first furosemide treatment and lasted for 48 h during days 6, 7 and 8. Blood was collected on the morning of day 7 (from 10.30 to 11.45 am, 2 to 3 h after the morning treatment) in the same animal ranking order as day 2. A 10 day washout interval was allowed between the last administration of furosemide and collection of the baseline blood sample in the next period.

### Blood sampling, processing and assays

Packed cell volume was measured before sample centrifugation (10 min, 1500 x ***g***, 20 °C) and storage of plasma aliquots at −80 °C until analysis. Plasma renin activity (Gamma Coat®, CA1553, DiaSorin Ltd, Bracknell, UK) was measured by radioimmunoassay, as validated by Syme et al. [34]. A radioimmunoassay validated in cats [30] was used to measured plasma aldosterone concentration (Coat-a-Count®, Siemens Medical Solutions Diagnostics, Los Angeles, CA, USA). Creatinine (Jaffé colorimetric method), protein, sodium, potassium, chloride and calcium (ILAB 600, Idexx, Warrington, UK) concentrations were measured at a commercial laboratory.

### Urine sampling, processing and assays

Urine was collected twice daily (08.00 am, 07.00 pm). Urinary specific gravity as recorded for each sample. Urine collected during the first day was stored at 4 °C for 24 h and pooled with urine from the second day. Accurate urination times and volumes were recorded to calculate 24 h averaged urinary volumes and analyte excretions over the collection period [33]. The 24 h urine excretion was corrected by the average percentage of voided volume lost through retention in the litter tray [33] and used alongside plasma and urinary creatinine concentrations to calculate GFR by endogenous urinary creatinine clearance. A non-selective COX inhibitor (ibuprofen 10 μM, Ref I7378, Sigma-Aldrich Co. Ltd, Poole, UK) solubilised in methanol was added to collected urine (1:1000 *v/v*) to prevent *ex vivo* eicosanoid production. Urine was centrifuged (1000 ***g***, 20 °C, 10 min) and the supernatant stored at −80 °C. Urinary electrolytes (sodium, chloride, potassium, calcium) were measured using ion sensitive electrodes and creatinine and protein concentrations were measured using Jaffé and Lowry methods, respectively (ILab 600 analyser, Idexx). Urinary aldosterone concentration was measured by RIA after liquid extraction with dichloromethane. Urinary prostanoids were co-extracted on reverse phase extraction columns (Varian SepPak 200 mg C18 LRC, Agilent) following a protocol adjusted from the manufacturer’s recommendation. Urinary TxB_2_ concentration was measured by RIA (TxB_2_ antibody, Ref P7291, Sigma and TxB_2_ Tracers, GE Healthcare biosciences Ltd, Little Chalfont, UK) and urinary PGE_2_ and 6-keto-PGF_2α_ were measured by enzymatic immunoassay (Immunoassays ADI-900-001 and ADI-900-004, Assay Designs, MI, USA). Urinary activity of γ-glutamyl transferase was measured on fresh samples within 24 h after the last collection using a colorimetric method (γ-GT, Randox, Crumlin, UK). Urinary NAG was measured with a commercially available colorimetric assay (N-acetyl-β-glucosaminidase kit, no. 875406, Roche, Basel, Switzerland), validated in the cat [31]. Details of all assay protocols and validation results for blood and urine samples are presented in the Additional file 2: S1.

### *Post-mortem* study and specimen provenance

*Post-mortem* kidney specimens were obtained (with informed owner consent) from 10 young (<5 years) and 11 old (>9 years) client-owned cats with normal renal function that had been euthanized or had died of natural non-renal causes. None of the cats had clinical signs indicative of chronic kidney disease (CKD), such as urinary specific gravity <1.035 or plasma creatinine >177 μmol L^−1^.

### Immunohistochemistry protocols

Three micrometre thick paraffin sections were dewaxed and rehydrated in grading ethanol followed by heating slides in a microwave at medium-high power in citrate buffer pH 6.0 for 15 min. Endogenous peroxidase activity was blocked by treating the tissue section with 3 % hydrogen peroxide for 10 min. Subsequently, 1.5 % normal goat serum (Vector Laboratories, Burlingame, CA) or 1.5 % normal donkey serum (Santa Cruz Biotechnology, Dallas, TX) was applied to block non-specific background staining of COX-1 and COX-2 primary antibodies, respectively. Slides were incubated with COX-1 (160108, Cayman Chemical, Camdridge, UK) or COX-2 (sc-1745, Santa Cruz Biotechnology) primary antibodies at 1:100 dilution at room temperature for 2 h and were then treated with biotinylated secondary antibody for COX-1 (BA-1000, Vector Laboratories) or COX-2 (sc-2023, Santa Cruz Biotechnology) for 1 h. Immunoactive complexes were detected by incubating the sections with avidin-biotin complex system (Santa Cruz Biotechnology) for 30 min and visualized with diaminobenzidine. The sections were counterstained with hematoxylin. Negative control slides were prepared by replacing the primary antibody with 1.5 % normal serum. Feline normal bladder sections were used as the COX-1 positive control [35], while canine foetal kidney sections were selected as the COX-2 positive control [36]. Stained sections were evaluated microscopically in a blinded manner and standard morphological criteria were used to identify the kidney structures. For each renal structure, staining intensity was assessed from 0 to 3. In each cat, 10 MD, 10 tubules and 20 glomeruli were randomly assessed and the median [interquartile range] intensity score was calculated as previously described [37].

### Statistical analysis

SAS software (Version 9.1.3, SAS Institute, Cary, NC, USA) was used for statistical analysis. When necessary, data were log transformed to satisfy the assumption of normal distribution of the residuals (Shapiro Wilk test) and homoscedasticity. An ANOVA model, containing sequence, period, sex, and baseline value as fixed effects and animal as a random effect, was fitted to all dependent variables. The effects of NSAID treatments were compared in a pairwise manner. The reported *p*-values are raw *p*-values corrected with a Tukey adjustment for multiple comparisons. In addition, the difference from baseline (or logarithmic equivalent) after furosemide for each NSAID treatment was tested against zero using a paired Student’s *t*-test. Results are presented as mean ± SD and *P* < 0.05 was considered significant. For immunostaining studies, the Kruskal-Wallis test was used to compare the intensity scores between groups with significance level set at *P* < 0.05.

## Conclusions

The present study indicates the lack of effect of acute COX inhibition on furosemide-induced diuresis and natriuresis in cats. It is suggested both COX-1 and COX-2 are involved in the PRA and aldosterone response to furosemide and that COX-2 may be involved in regulating pathways other than angiotensin II stimulated aldosterone secretion. These findings, supported by the immunohistochemical localisation of COX-1 in the MD of the feline kidney, illustrate that renal physiology is species-dependent.

## Additional files

Additional file 1 S1:
**PK-PD integration of robenacoxib and ketoprofen (description of data): estimation of the average duration of COX-1 and COX-2 inhibition achieved with robenacoxib once a day (RSID), robenacoxib twice a day (RBID) and ketoprofen (KET) using data from the literature.** (DOCX 424 KB) Additional file 2 S2:
**analytical method description and validation: (description of data) Validation data for Plasma Renin Activity, plasma and urinary aldosterone, urinary prostanoids and urinary enzymes.** (DOCX 22 kb)

## Abbreviations

COX: cyclooxygenase
GFR: glomerular filtration rate
NSAIDs: non-steroidal anti-inflammatory drugs
MD: macula densa
JGA: juxtaglomerular apparatus
PRA: plasma renin activity
PGE_2_: prostaglandin E2
6-keto-PGF1_α_: 6-keto-prostaglandin F_1_alpha (stable metabolite of Prostacyclin)
TxB_2_: thromboxane B_2_
NAG: N-acetyl-beta-D-glucosaminidase
