# Supplementary material for: Effect of Cyclooxygenase(COX)-1 and COX-2 inhibition on furosemide-induced renal responses and isoform immunolocalization in the healthy cat kidney
Source: BMC Vet Res. 2015 Dec 3;11:296. doi: 10.1186/s12917-015-0598-z (PMC4669647; doi:10.1186/s12917-015-0598-z)
Supplement: Additional file 1 S1: — PK-PD integration of robenacoxib and ketoprofen (description of data): estimation of the average duration of COX-1 and COX-2 inhibition achieved with robenacoxib once a day (RSID), robenacoxib twice a day (RBID) and ketoprofen (KET) using data from the literature. (DOCX 424 KB) [file 12917_2015_598_MOESM1_ESM.docx]

**Additional file 1:**

**(title) S1 PK-PD integration of robenacoxib and ketoprofen**

**(description of data): estimation of the average duration of COX-1 and COX-2 inhibition achieved with robenacoxib once a day (RSID), robenacoxib twice a day (RBID) and ketoprofen (KET) using data from the literature:**

In many studies, the lack of PK/PD integration comparing circulating NSAID concentrations and COX-1 and COX-2 potencies (IC_50_) may lead to erroneous conclusions regarding selectivity of inhibition. It is indeed possible for a COX-1 selective drug, such as ketoprofen, to inhibit COX-2 clinically as demonstrated below. The average pharmacokinetic profiles can be simulated for robenacoxib (at doses of RSID, RBID, Fig 6.a) and S(+) ketoprofen (Fig 6.b) (Lees, Taylor et al., 2003; Schmid, Seewald et al., 2010; King, Hotz et al., 2012; Pelligand, King et al., 2012). Robenacoxib and ketoprofen COX inhibition potency data (PD), derived from *in vivo* and *ex vivo* feline assays, were used for the PK/PD integration. Robenacoxib is COX-2 selective with median IC_50_ COX-2 values ranging from 14.1 to 38.2 ng mL^-1^ (Pelligand, King et al., 2012) and median IC_50_ COX-1 of 2415 ng mL^-1^. The S(+) KTP enantiomer is COX-1 selective with median IC_50_ COX-2 of 48.5 ng mL^-1^ and median IC_50_ and IC_90_ COX-1 of 0.45 and 12.3 ng mL^-1^, respectively (Pelligand, King et al., 2014). For robenacoxib, average blood concentration is predicted to exceed the IC_50_ COX-2 for 4.2 to 5.7 h d^-1^ for RSID and 8.4 to 11.4 h d^-1^ for RBID (Fig 6.a). There is virtually no inhibition of COX-1 with RSID and RBID, as peak blood concentration never attained IC_50_ COX-1. For S(+) KTP, plasma concentrations exceeded IC_90_ COX-1 for 24 h (complete inhibition) and IC_50_ COX-2 for 10 h after the first administration and 11.8 h after the second administration (Fig 6.b). Therefore, interpretation of the data is complicated by the fact that KTP (at the clinical dose rate and route necessarily selected) inhibits not only COX-1 for 24 h, but also COX-2 for the same cumulated duration as RBID.


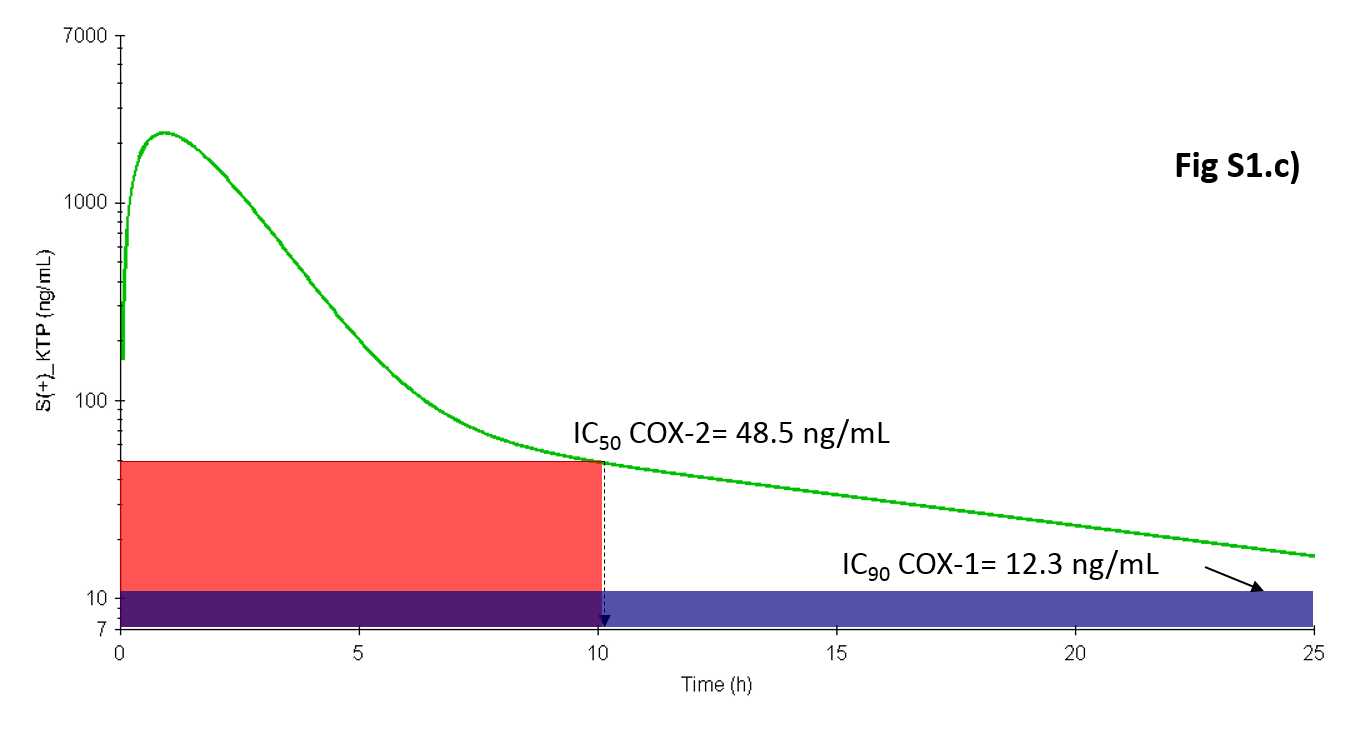

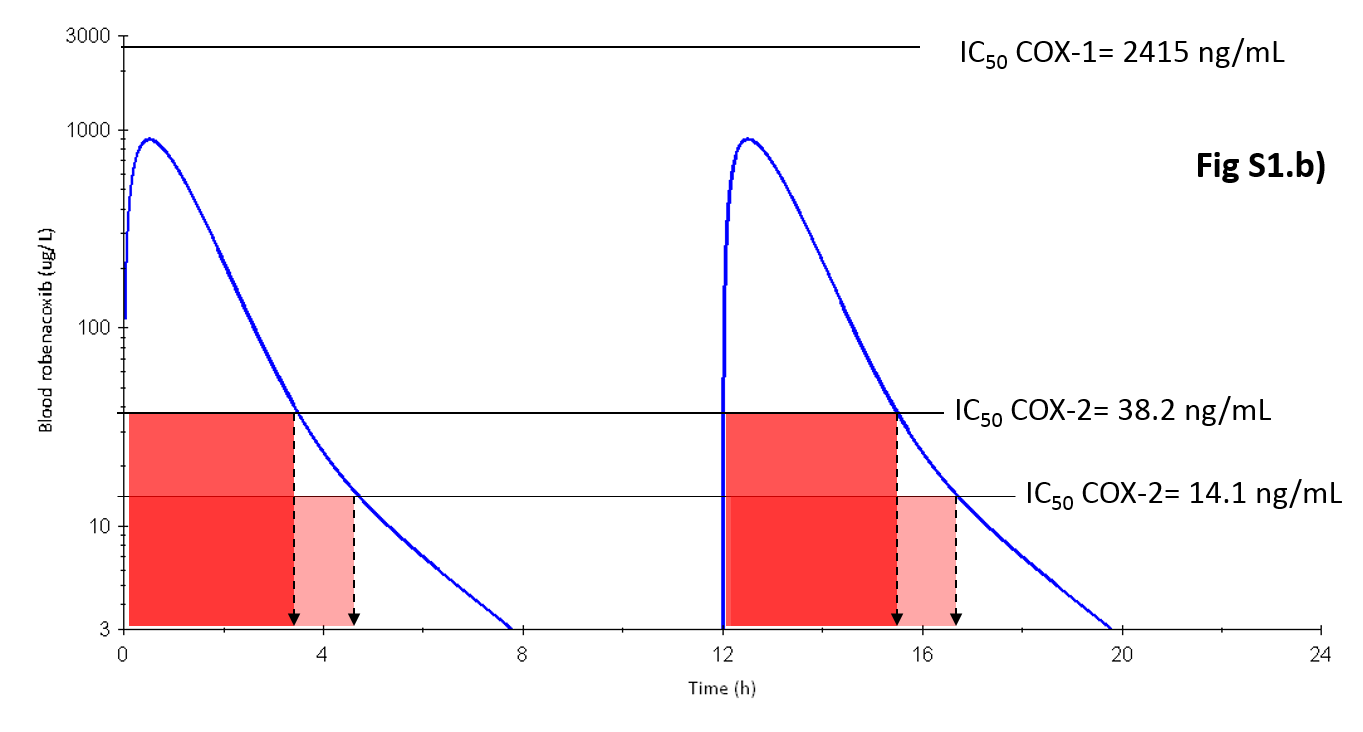

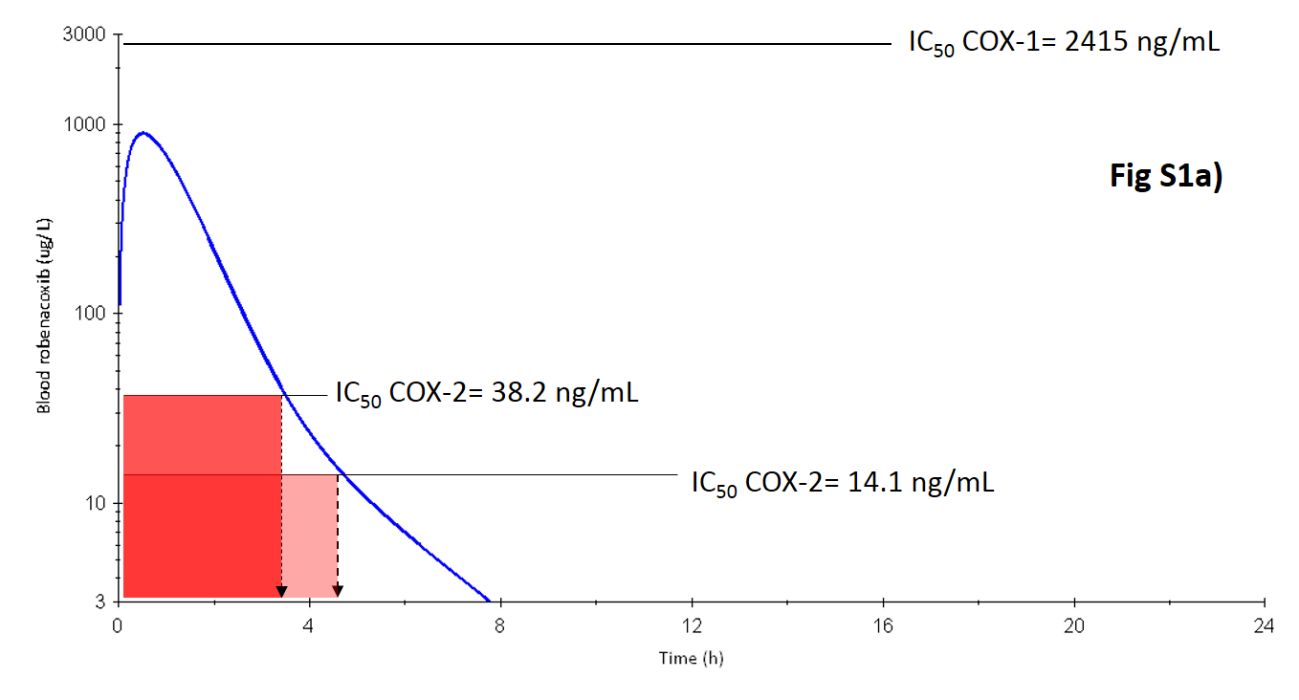


Figure S1.a: Simulated blood robenacoxib concentration-time profile (blue curve) from Pelligand et al. 2012, 2014 and Schmid et al. 2010, King et al. 2010 after single oral administration of a 1 mg kg^-1^ dose (average profile obtained with data from 25 cats receiving 1.6 ±0.24 mg kg^-1^) and corresponding IC_50_ COX-1 and IC_50_ COX-2. Red area, average time during which plasma concentration is above IC_50_ COX-2 (shade of red depending on IC_50_ estimates).

Figure S1.b: Simulated blood robenacoxib concentration-time profile (blue curve) from Pelligand et al. 2012, 2014 and King et al. 2010 after repeated oral administration (1 mg kg^-1^ BID).

Figure S1.c: Simulated plasma S(+) ketoprofen concentration-time profile (green curve) from Lees et al. 2003 after single oral administration (racemic KTP, 1 mg/kg, n=6 cats) and corresponding IC_50_ COX-2 (Pelligand et al. 2012, 2014). Red area, average time during which plasma concentration is above IC_50_ COX-2. Blue area, time during which plasma concentration exceeds IC_90_ COX-1 (> 24h).

**References:**

Lees, P., Taylor, P.M., Landoni, F.M., Arifah, A.K. & Waters, C. (2003) Ketoprofen in the cat: pharmacodynamics and chiral pharmacokinetics. The Veterinary Journal, 165(1), 21-35.

Schmid, V.B., Seewald, W., Lees, P. & King, J.N. (2010) In vitro and ex vivo inhibition of COX isoforms by robenacoxib in the cat: a comparative study. Journal of Veterinary Pharmacology and Therapeutics, 33(5), 444-452.

King, J.N., Hotz, R., Reagan, E.L., Roth, D.R., Seewald, W. & Lees, P. (2012) Safety of oral robenacoxib in the cat. J Vet Pharmacol Ther, 35(3), 290-300.

Pelligand, L., King, J.N., Toutain, P.L., Elliott, J. & Lees, P. (2012) PK-PD modelling of robenacoxib in a feline tissue cage model of inflammation. Journal of Veterinary Pharmacology and Therapeutics, 35(1), 19-32.

Pelligand, L., King, J.N., Hormazabal, V., Toutain, P.L., Elliott, J. & Lees, P. (2014) Differential pharmacokinetics and pharmacokinetic/pharmacodynamic modelling of robenacoxib and ketoprofen in a feline model of inflammation. J Vet Pharmacol Ther, 37(4), 354-366.
