# Supplementary material for: Effect of Cyclooxygenase(COX)-1 and COX-2 inhibition on furosemide-induced renal responses and isoform immunolocalization in the healthy cat kidney
Source: BMC Vet Res. 2015 Dec 3;11:296. doi: 10.1186/s12917-015-0598-z (PMC4669647; doi:10.1186/s12917-015-0598-z)
Supplement: Additional file 2 S2: — analytical method description and validation: (description of data) Validation data for Plasma Renin Activity, plasma and urinary aldosterone, urinary prostanoids and urinary enzymes. (DOCX 22 kb) [file 12917_2015_598_MOESM2_ESM.docx]

**Additional file 2:**

**(title) S2 analytical method description and validation:**

**(description of data) Validation data for Plasma Renin Activity, plasma and urinary aldosterone, urinary prostanoids and urinary enzymes.**

**S2.a: Plasma Renin Activity measurement: (previously validated by Syme et al. (**[**2003**](#_ENREF_40)**)**

*Assay method*:

The Plasma Renin Activity kit involves an initial incubation of plasma to generate angiotensin I followed by quantification of angiotensin I by a competitive radioimmunoassay. A volume of 0.5 mL of plasma is transferred to an uncoated tube containing a generation buffer with optimal pH and an inhibitor of conversion of Angiotensin I in angiotensin II. Half of the sample is maintained at 4° C in an ice bath and the other half is incubated at 37° C for 90 min and then placed in the ice bath. The assay was carried out exactly according to the manufacturer’s instructions.

The amount of angiotensin I was measured in duplicate for both incubation temperatures. A volume of 100 µL of sample or calibrator and 1 mL of ^125^I Angiotensin I tracer were transferred in a tube coated with antibodies directed against Angiotensin I and mixed. After 3 h incubation, the tubes were decanted and counted in a gamma counter for 1minute. The standard curve ranged from 0 to 5.0 ng/0.1mL of Angiotensin I and was produced in excel with the percent bound plotted against log concentration. Unknown values were then calculated by interpolation on the standard curve and the amount generated at 4° C was subtracted from the amount generated at 37° C.

*Intra and inter-assay coefficient of variations:*

Intra and inter assay coefficients of variations (CV) were calculated from replicate measurements of Angiotensin I generated by a quality control plasma sample included in the kit. For the furosemide trial, intra assay CV was 5.1 to 9.3% and inter assay CV was 9.6%.

**S2.b: Aldosterone measurement (**[**Syme, Fletcher et al., 2007**](#_ENREF_41)**):**

*Plasma aldosterone extraction:*

A volume of 450 µL of sample was placed in a glass tube with 10 µL of ^3^H Aldosterone and 10 mL of dichloromethane. The tube was capped, vortex mixed and the aqueous supernatant aspirated. The organic phase was evaporated at 37° C under a stream of nitrogen and reconstituted in the assay kit zero standard (500 µL) and vortex mixed. A 50 µL aliquot was counted in a beta counter to calculate aldosterone recovery and correct for the losses due to the extraction process. A multivalent plasma quality control (QC, low, medium and high concentrations) was inserted in the batch to calculate intra and inter-assay CV.

*Urine aldosterone extraction:*

Urine samples (500 μl) were subjected to solvent extraction by the addition of 5 ml methyl formate, the tubes were capped and placed in a rotary mixer and mixed at 30 r.p.m. for 60 min. Tubes were then centrifuged at 600 ***g*** for 5 min and 500 µL of supernatant was evaporated to dryness at 37° C under a stream of nitrogen. The residues were reconstituted in the assay kit zero standard (250 μl) and vortex-mixed.

*Assay method:*

Aldosterone concentration was then measured by competitive binding radio-immunoassay (RIA) using a commercially available RIA kit for human plasma and urine (Coat-a-count Aldosterone, Diagnostic Products Corporation, California, USA). The assay was carried out exactly according to the manufacturer’s instructions. The standard curve ranged from 0 to 1200 pg/mL of aldosterone and was produced in excel with the percent bound plotted against log concentration. Unknown values were then calculated by interpolation on the standard curve. The results obtained from the calibration curve were then corrected for the dilution factor associated with extraction and reconstitution.

*Intra and inter-assay coefficient of variations:*

Intra assay CV ranged from 4.0 (high concentration QC) to 15.6% (low concentration QC) %. Inter assay variability was between 2.2 (medium concentration QC) and 10.2% (low concentration QC).

*Extraction recovery:*

The plasma aldosterone extraction recovery was 54.1 ±8.0%. Urinary recoveries were not calculated.

**S2.c: Urinary TxB2, and PGE2 extraction and recovery from cat urine:**

*Co-extraction procedure:*

The silica of the SPE cartridges was activated with 5 mL of methanol followed by 5 mL of deionised water. 0.5 mL of sample was diluted with the same volume of water. The sample was acidified to pH approx. 3.0 by addition of 2 M HCl and left at 4º C for 15 min before centrifugation at 2000 ***g*** for 10 min. The supernatant was applied to the column under and left filter down by gravity (without using positive or negative pressure). The column was washed with 5 mL of deionised water and 5 mL of hexane, before allowing the silica to dry for 15 min with high negative pressure. The samples were eluted with 5 mL of 1% methanol in methyl formate (v/v) and the eluate collected in new glass tubes. The eluate was vaporated at 50º C under a stream of nitrogen in a sample concentrator. The sample was reconstituted with 0.25 mL of RIA buffer and vortex-mixed thoroughly three times at 5 min intervals. The extracted samples were therefore concentrated 2-fold.

Urinary TxB_2_ was measured without dilution. Urinary PGE_2_ and PGI_2_ were measured after a 4-fold or a 8-fold dilution (exceptionally after a 12-fold dilution)

*Assays procedure:*

The PGE_2_ and PGI_2_ assay was carried out according to the manufacturer recommendations. The methods for TxB_2_ measurement in urine were adapted from previous publication ([Higgins & Lees, 1984](#_ENREF_13)). Lyophilised TxB_2_ antibody (Ref. P7291, Sigma Aldrich) was reconstituted in 15 mL of assay buffer. One µg/mL TxB_2_ solutions were prepared from the standards obtained commercially (Ref. T0516 for TxB_2_ standards, Sigma Aldrich). The solution was subsequently diluted in assay buffer to yield eight working standard concentrations 0.05, 0.1, 0.2, 0.5, 1, 2, 5 and 10 ng/ml. The eight concentrations were used to prepare the standard curves. The ^3^H TxB_2_ tracer (GE Healthcare biosciences Ltd, Little Chalfont, UK) were diluted 1:1000 in assay buffer. Total activity, non-specific binding and total binding were evaluated at the beginning of each batch. After adding tracer and antiserum, all tubes were vortexed and incubated at 4^o^ C for 16-24 h. Two hundred µL of dextran-coated charcoal suspension (dextran 70, GE healthcare) were added to all tubes except total counts. The tubes were then vortexed, incubated at 4^o^ C for 10 min and centrifuged at 1500 ***g*** for 10 min at 4^o^ C. The supernatant was decanted into scintillation vials in which 4 ml of scintillant were added (Scintillant LS-275, National Diagnostic, Hessle, UK). A standard curve was generated by plotting the normalized percentage bound as a function of the log_10_ TxB_2_ concentration. The sample concentrations were extrapolated from the standard curve plot. At least two concentrations of pooled samples were aliquoted and used as quality controls. They were dispersed over the sequences of unknown samples to calculate inter and intra-assay variability.

*Validation results:*

- Intra- inter- assay variabilities:

Urinary TxB_2_ intra assay variability was 9.0 to 20.1% for the high, 3.8 to 6.6% for the medium, 9 to 13.7% for the low concentration control.

Urinary TxB_2_ inter assay variability was 10.2% for the high, 6.3% for the medium and 8.0% for the low concentration control.

PGE_2_ intra assay variability was 17.5% for the high, 5.8% for the medium and 8.9% for the low concentration control.

- Recoveries

PGE_2_, TxB_2_ and 6k PGF1a extraction recoveries were 87 ±4.8, 89 ±3.8 and 65 +/- 2.6%, respectively.

- PGE_2_ stability at room temperature:

There was no significant difference in PGE_2_ concentration when measured on fresh urine (0h), urine kept 12 h in the reservoir at room temperature (12 h) and kept 12 h at room temperature and 24 h in the fridge at 4° C (12 h + 24 h) (Dunn's Multiple Comparison Test, P = 0.43).

- Antibody cross-reactivities:

PGE_2_ assay cross reactivity with was 0.6% with 6-ketoPGF1α and less than 0.1% with TxB_2_. There was no information available from the PGE_2_ kit manufacturer regarding cross reactivity with PGE-M.

Urinary excretion of 2,3 dinor TxB_2_ reflects total body thromboxane synthesis, whereas urinary TxB_2_ reflects renal production (see chapter 1). Cross-reactivity of the TxB_2_ antibody for 2,3 dinorTxB_2_ was 16.4%. Cross reactivity for PGE_2_, 6keto PGF1α were negligible (less than 0.1%).

Urinary excretions of 2,3 dinor 6keto PGF1a and 6-ketoPGF1a reflect total body synthesis whereas renal prostacyclin is excreted as 6keot PGF1a exclusively ([Stichtenoth, Marhauer et al., 2005](#_ENREF_38)) Cross reactivity for 2,3 dinor PGF1a was 3.17%.

**S2.d: Urinary gamma-Glutamyl transpeptidase (γ-GT) and N-acetyl-beta-D-glucosaminidase (NAG) measurement in cat urine:**

Intra-assay variability for urinary γ-GT measurement assessed with high and low human serum controls was less than 10.6% and inter-assay variability was less than 7.7%. Inter-assay variability for the urinary NAG assay ranged from 4.4 to 8.5%.
